# Evolutionary Comparison of the Chloroplast Genome in the Woody *Sonchus* Alliance (Asteraceae) on the Canary Islands

**DOI:** 10.3390/genes10030217

**Published:** 2019-03-14

**Authors:** Myong-Suk Cho, Ji Young Yang, Tae-Jin Yang, Seung-Chul Kim

**Affiliations:** 1 Department of Biological Sciences, Sungkyunkwan University, Suwon 16419, Korea; marina0426@gmail.com; 2 Research Institute for Ulleung-do and Dok-do Island, Kyungpook National University, Daegu 41566, Korea; whity@daum.net; 3 Department of Plant Science, Plant Genomics and Breeding Institute, Research Institute of Agriculture and Life Sciences, Seoul National University, Seoul 08826, Korea; tjyang@snu.ac.kr

**Keywords:** woody *Sonchus* alliance, Asteraceae, oceanic endemic species, adaptive radiation, insular woodiness, chloroplast genome evolution, comparative genomic analysis

## Abstract

The woody *Sonchus* alliance consists primarily of woody species of the genus *Sonchus* (subgenus *Dendrosonchus*; family Asteraceae). Most members of the alliance are endemic to the oceanic archipelagos in the phytogeographic region of Macaronesia. They display extensive morphological, ecological, and anatomical diversity, likely caused by the diverse habitats on islands and rapid adaptive radiation. As a premier example of adaptive radiation and insular woodiness of species endemic to oceanic islands, the alliance has been the subject of intensive evolutionary studies. While phylogenetic studies suggested that it is monophyletic and its major lineages radiated rapidly early in the evolutionary history of this group, genetic mechanisms of speciation and genomic evolution within the alliance remain to be investigated. We first attempted to address chloroplast (cp) genome evolution by conducting comparative genomic analysis of three representative endemic species (*Sonchus acaulis*, *Sonchus canariensis*, and *Sonchus webbii*) from the Canary Islands. Despite extensive morphological, anatomical, and ecological differences among them, their cp genomes were highly conserved in gene order and content, ranging from 152,071 to 152,194 bp in total length. The number of repeat variations and six highly variable regions were identified as valuable molecular markers. Phylogenetic analysis of 32 species in the family Asteraceae revealed the phylogenetic position of the woody *Sonchus* alliance within the tribe Cichorieae and the sister relationship between the weedy *Sonchus oleraceus* and the alliance.

## 1. Introduction

The current, redefined genus *Sonchus* (Asteraceae) in its wider circumscription is comprised of ca. 95 species, consisting of the subgenera *Dendroseris*, *Dendrosonchus,* and *Origosonchus* and other widely distributed weedy species that are tentatively classified under the subgenus *Sonchus* [1,2]. *Sonchus* is widely distributed, extending from the Mediterranean region to the mid-Atlantic islands, temperate Eurasia, tropical Africa, Australia/New Zealand, North America, and the South Pacific Juan Fernández and Desventuradas Islands [1]. While the subgenus *Dendroseris* includes the endemic species of the Pacific islands distributed in the archipelagos of Juan Fernández and Desventuradas, the subgenus *Origosonchus* is mainly distributed in Africa, with some species also occurring in Asia (Saudi Arabia and Yemen). The subgenus *Dendrosonchus* consists of approximately 35 woody species, known as the woody *Sonchus* alliance, distributed in the Macaronesian Islands of the Atlantic Ocean, including the archipelagos of the Canaries, Madeira, and Cape Verde. Two taxa, *Sonchus webbii* and *Sonchus tuberifer*, are the only members of the alliance that do not have a true woody habit; they are herbaceous perennials with tuberous roots. The entire alliance is endemic to the Macaronesian Islands, with the exception of one species, *Sonchus pinnatifidus,* occurring in both the Canaries and western Morocco. Specifically, all but four species of the woody *Sonchus* alliance are endemic to the Canaries [3]. The Canary archipelago consists of seven main islands and several small islets. These islands, which are of volcanic origin and diverse geological ages ranging from 0.8 to 20 million years, display a rich flora comprised of 570 endemic plant species [4], a high percentage (72%) of which is constituted of woody endemics [5].

Adaptive radiation on oceanic islands has yielded spectacular and explosive in situ diversification, often resulting in significant divergence from the common habits of the corresponding taxonomic relatives occurring on the continents [6]. The woody *Sonchus* alliance has been the subject of intensive evolutionary studies, as it represents the most outstanding example of the adaptive radiation and insular woodiness on oceanic islands. Previous studies demonstrated the monophyly of the entire alliance based on both nuclear ribosomal DNA (nrDNA) and chloroplast DNA (cpDNA) phylogenies with strong bootstrap support [3,7,8,9,10,11,12], even though the members of the alliance display great morphological, ecological, and anatomical diversity. This implies that all taxa in the alliance were derived from a single herbaceous colonizer from continent; the extraordinary diversity evolved in situ in the Macaronesian islands most likely originated from the extensive radiation process and adaptation to a wide diversity of habitats within the archipelagos. During adaptive radiation, the trend towards increased woodiness of woody *Sonchus* could have been favorable for colonizing and adapting to the diverse habitats in the Macaronesian Islands. Carlquist [6] suggested that the endemic frutescent species found on many oceanic islands are the result of an increase in woodiness in response to the uniformity of insular climates, and that the insular woody life-forms represent a derivation from the herbaceous life-form of the ancestors. Mapping of the growth-form traits based on internal transcribed spacer (ITS) of nrDNA and cpDNA phylogeny supported Carlquist’s hypothesis, i.e., the herbaceous origin of the woody *Sonchus* alliance, rejecting the suggestion of a relictual nature of the ancient lineages [7,8,11]. As those phylogenies show a general trend towards increased woodiness, it is likely that the ancestor of the entire alliance was an herbaceous perennial, with evolution toward caudex perennials, shrubs, and trees of different lineages occurring during the radiation in the Macaronesian Islands. This trend, e.g., insular endemics, predominantly herbaceous plants, that have evolved woodiness and developed tree-or shrub-like habit on different islands, is a well-known convergent feature in numerous genera, including *Sonchus*, *Echium*, *Argyranthemum*, *Pericallis*, and *Crambe* of the Macaronesian endemics.

However, the closest continental relatives of the woody *Sonchus* alliance are still elusive, as the phylogenetic position of the alliance within the subtribe Sonchinae and closest continental relatives was weakly supported or lacked enough resolution despite its robust monophyly. Specifically, cpDNA phylogeny did not have enough resolution to identify any apparent continental sister group, while nrDNA ITS phylogeny suggested that the alliance evolved from a common ancestor shared with the western European herbaceous perennial *Sonchus palustris* or with the Iberian/Moroccan endemic small suffrutescent perennial *Sonchus* section *Pustulati* albeit low support value (i.e., BS < 50%) [3,7,8,9,10,11,12]. This incongruence between nuclear and chloroplast phylogenies could be the result of the differences for rate heterogeneity between two genomes (i.e., too slow and two fast in cpDNA coding region and nrDNA ITS, respectively), and further cpDNA phylogeny was not sufficient to reconstruct the rapid radiation events of the major lineages in the alliance based on several coding and noncoding regions only. Genetic linkage analysis and subsequent quantitative trait loci (QTL) mapping study were also carried out to dissect the genetic basis of insular woodiness using two species on the Canary Island, *Sonchus radicatus* with a thick woody stem and a herbaceous perennial, *S. webbii*. The results suggested that the woody habit appeared to be under simple genetic control, but no significant QTLs were detected [13,14].

**Figure 1:**
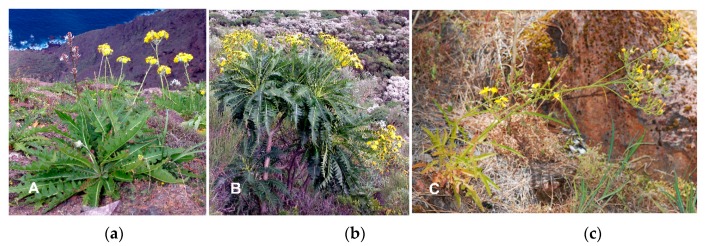
Photographs of three representative species of the woody *Sonchus* alliance in the Canary Islands. (**a**) *Sonchus acaulis*, a caudex perennial (photo credit: Seung-Chul Kim); (**b**) *Sonchus canariensis*, a tall shrub (photo credit: Seung-Chul Kim); (**c**) *Sonchus webbii*, a tuberous herbaceous perennial (photo credit: Aurelio Acevedo).

As an attempt to better understand the origin of the woody *Sonchus* alliance and its woodiness, we characterized the complete chloroplast genomes of three *Sonchus* species in the alliance; two woody perennials with different life forms (*Sonchus acaulis*, a caudex perennial, and *Sonchus canariensis*, a tall shrub or small tree) and one herbaceous perennial (*S. webbii*). These three *Sonchus* species show extensive morphological, anatomical, and ecological divergence; *S. acaulis* has a woody base with leaves in a single, large basal rosette up to 1 m diameter, while *S. canariensis* is a tall, upright shrub growing up to 3 m height, bearing pinnatisect leaves with 10–15 pairs of equally spaced lobes. *S. webbii* is an herbaceous perennial with tuberous roots and leafy stem of up to 30 cm length (Figure 1). Their ecological niches and distribution in the Canary Islands also differ from each other. Both *S. acaulis* and *S. canariensis* occur in relatively old islands of Tenerife (11.6 million years (myr) old) and Gran Canaria (14–16 myr old), but *S. acaulis* is widely spread in the forests and xerophytic zones where *S. canariensis* is very rarely found. *Sonchus webbii* is also rare but is highly restricted to the northern part of La Palma, a young island (2 myr old).

Chloroplasts in plant cells play a crucial role in sustaining life on earth by converting solar energy to carbohydrates through the process of photosynthesis and oxygen release. They encode many key proteins that are involved in photosynthesis and other metabolite syntheses [15]. The phylogenetic studies of several plant families have been greatly facilitated by deployment of chloroplast DNA markers to resolve the evolutionary relationship within phylogenetic clades [15]. However, the partial chloroplast phylogeny based on several coding and noncoding cpDNA regions in previous studies has not provided enough resolution to identify an apparent continental sister group to address the origin of the woody *Sonchus* alliance [11]. Since the advent of next-generation sequencing (NGS) methods, whole chloroplast genome sequencing has facilitated faster and cheaper methods to sequence whole chloroplast genomes and increase phylogenetic resolution at lower taxonomic levels in plant phylogenetic and population genetic analyses [16]. The benefits of genome-wide data have improved our understanding of plant evolution and diversity in the field of chloroplast genetics and genomics, particularly in the lineages with previously unresolved relationships [15]. In the present study, we conducted a comparative genomic analysis among three diverse species of woody and herbaceous perennials to gain first insight into chloroplast genome evolution in the woody *Sonchus* alliance in the Canary Islands. The chloroplast genome has never been characterized in the plant endemics to the Macaronesian Islands.

## 2. Materials and Methods

### 2.1. Material Preparation, DNA Extraction, Genome Sequencing, and Annotation

**Figure 2:**
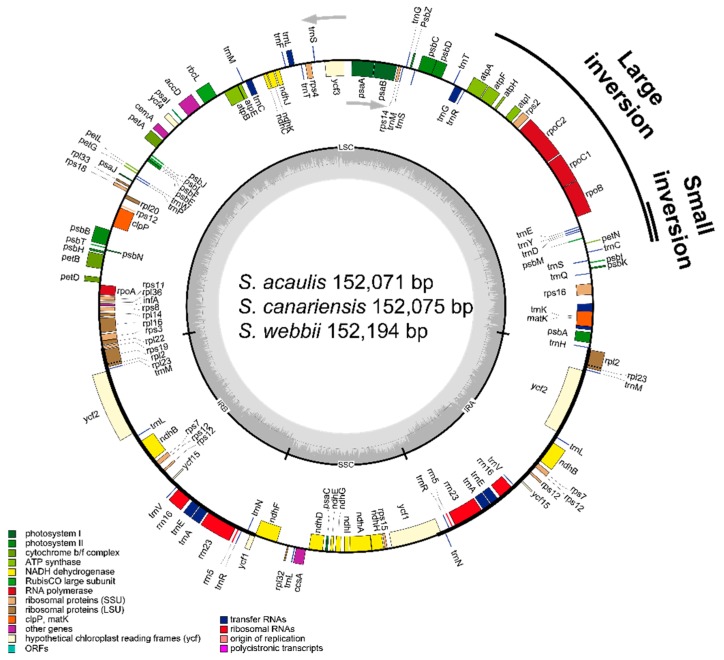
Gene map of three *Sonchus* species. The genes inside and outside of the circle are transcribed in the clockwise and counterclockwise directions, respectively. Genes belonging to different functional groups are shown in different colors. The thick lines indicate the extent of the inverted repeats (IR_A_ and IR_B_) that separate the genomes into small single copy (SSC) and large single copy (LSC) regions. Large inversion and smaller inversion nested within the large inversion are indicated with black lines outside of the gene map.

The silica-gel dried leaves sampled from natural habitats in the Canary Islands, Spain were used as sources of DNA. Total genomic DNA was isolated using the DNeasy Plant Mini Kit (Qiagen, Carlsbad, CA, USA). An Illumina paired-end (PE) genomic library was constructed and sequenced using the Illumina HiSeq platform according to the standard Illumina PE protocol. The sequence reads were assembled by using a CLC genome assembler (ver. 4.06 beta, CLC Inc, Aarhus, Denmark) with coverage of 256.85× for *S. acaulis*, 223.30× for *S. canariensis*, and 158.08× for *S. webbii*. Annotation was performed with the Dual Organellar GenoMe Annotator [17], ARAGORN v1.2.36 [18], and RNAmmer 1.2 Server [19]. Using Geneious v8.1.6 (Biomatters Ltd., Auckland, New Zealand), the draft annotation was inspected and corrected manually by comparison with homologous genes in *Lactuca sativa* (DQ383816) and *Sonchus oleraceus* (MG878405) from the NCBI GenBank database. The completed sequences were registered in GenBank under accession numbers MK033506 (*S. canariensis*), MK033507 (*S. acaulis*), and MK033508 (*S. webbii*). OGDRAW [20] was used to draw a circular chloroplast genome map (Figure 2).

### 2.2. Repeat Sequence Analysis

REPuter [21] was used to detect the repetitive structure of the three *Sonchus* chloroplast genomes and locate various types of repeat sequences for forward, reverse, complement, and palindromic match directions. Search parameters were set to: maximum computed repeats = 50, minimum repeat size = 8 bp, and hamming distance = 0. Simple sequence repeats (SSRs) were identified using MISA web (http://pgrc.ipk-gatersleben.de/misa/) with search parameters of 1–15 (unit size-minimum repeats, i.e., mono-nucleotide motifs with 15 minimum numbers of repetition), 2–5, 3–3, 4–3, 5–3, and 6–3 with 0 interruption (maximum difference for two SSRs).

### 2.3. Identification of Highly Divergent Regions

The three *Sonchus* chloroplast genomes were compared at the entire chloroplast genomic level using DnaSP [22] and mVISTA [23]. Overall sequence divergence was investigated for sequence similarities and differences, with the two species of *S. canariensis* and *S. webbii* aligned and compared to *S. acaulis* using the LAGAN alignment mode [24] in mVISTA. Nucleotide diversity was calculated by using the sliding window analysis (window length = 1000 bp and step size = 200 bp excluding sites with alignment gaps) to detect the most divergent regions among the three *Sonchus* species in DnaSP. The borders of large single copy (LSC), small single copy (SSC), and inverted repeats (IRs) regions were compared with the results of DnaSP and mVISTA.

### 2.4. Phylogenetic Analysis

To investigate the taxonomic position and phylogenetic relationship of the newly sequenced three species of the woody *Sonchus* alliance, 29 complete chloroplast sequences representing Asteraceae species were downloaded from GenBank. A total of 32 species, including these three species, were aligned using MAFFT v.7 [25]. A maximum likelihood (ML) tree was produced based on the relationships of whole chloroplast genomes by IQ-TREE [26] with 1000 replicate bootstrap (BS) analyses. The best fit evolutionary model was chosen as TVM + F + I + G4, scored according to the Bayesian information criterion (BIC) scores and weights by using ModelFinder [27] implemented in IQ-TREE.

## 3. Results and Discussion

### 3.1. Comparative Genome Analysis of Three Sonchus Species in Content, Order, and Organization

**Figure 3:**
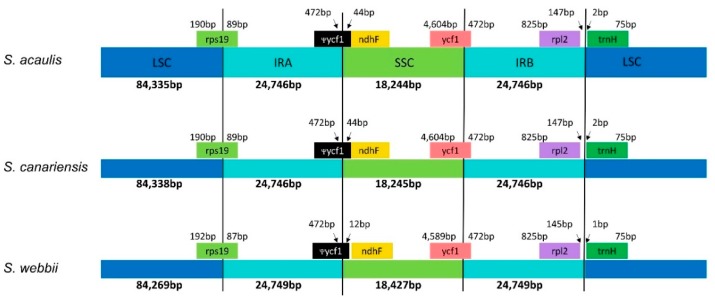
Comparison of the border positions of LSC, SSC, and IR regions among three chloroplast genomes of *Sonchus* species in the alliance. Gene names are indicated in boxes, and their lengths in the corresponding regions are displayed above the boxes.

Despite extensive morphological, anatomical, and ecological differences among three *Sonchus* species (i.e., *S. canariensis*, *S. webbii*, and *S. acaulis*), pairwise identity among their complete chloroplast genomes was strikingly high in sequence (99.6%), gene content, and organization. The size of three chloroplast genomes ranged from 152,071 (*S. acaulis*) to 152,194 (*S. webbii*) base pairs (bp), with only minor length differences among them, and consisted of four typical regions: LSC, SSC, and a pair of IR_A_ and IR_B_. One large inversion known as 22.8 kb and a second smaller inversion, 3.3 kb, nested within the large inversion were found in chloroplast genomes of all three *Sonchus* species (Figure 2). These two cpDNA inversions unique in Asteraceae are shared by all major clades of Asteraceae except members of subfamily Barnadesioideae distributed in Andes, South America, as reported in comparison with other outgroup species in Campanulaceae, Goodeniaceae, Ericaceae, Pittosporaceae and *Nicotiana tabacum* (Solanaceae), which do not have them [28,29]. The overall guanine-cytosine (GC) content of each chloroplast genome was 37.6%, with LSC, SSC, and IR regions having 35.8%, 31.5%, and 43.1% GC contents, respectively. All three *Sonchus* cp genomes contained 131 genes, including 88 protein-coding genes, six rRNA genes, and 37 tRNA genes. Nineteen genes contained introns, including nine tRNA genes. Three genes of *clp*P, *rps*12, and *ycf*3 exhibited two introns. The *trn*K tRNA gene harbored the largest intron, which contained the *mat*K gene. A total of 18 genes were duplicated in the inverted repeat regions, including seven tRNAs, three rRNAs, and eight protein genes. The trans-splicing gene *rps*12 consisting of 3 exons was located in the LSC region for exon 1, but exon 2 and exon 3 of the gene were imbedded in the IR regions. Part of *ycf*1 was duplicated in the IR_A_ region, forming a pseudogene (Figure 2 and Figure 3, Table 1 and Table 2).

### 3.2. Simple Sequence Repeats and Large Repeat Sequences

Microsatellites or SSRs represent a unique type of tandemly repeated genomic DNA sequences. They have high polymorphisms because of large variations in motifs and number of repetitions. Microsatellites range from one to six nucleotides in length, and are typically classified as mono-, di-, tri-, tetra-, penta-, and hexa-nucleotide repeats. The location of the microsatellites in the genome determines their functional role, allowing the potential to affect many aspects of genetic function, including gene regulation, development, and evolution. Because of the high level of polymorphisms and genome-wide distribution, microsatellite markers have been powerful tools in population genetics to measure genetic diversity and address population genetic issues at the level of inter- and intraspecific variations, such as gene flow, parentage, and population structure [30]. In spite of the nature of conservative chloroplast genome retaining low level of substitution rate, Powell et al. [31] reported that microsatellites identified in chloroplast genomes (cpSSRs) revealed extensive intraspecific variability to clarify phylogenetic relationships and to further determine the geographical distribution of genealogical lineages of *Glycine* species (soybeans). The occurrence of population-specific cpSSR polymorphisms have been also documented in other plants of Scots pine (*Pinus sylvestris* L.) [32], wheat (*Triticum* species) [33], European silver fir (*Abies alba* Mill.) [34], and *Cucumis* species [35].

**Figure 4:**
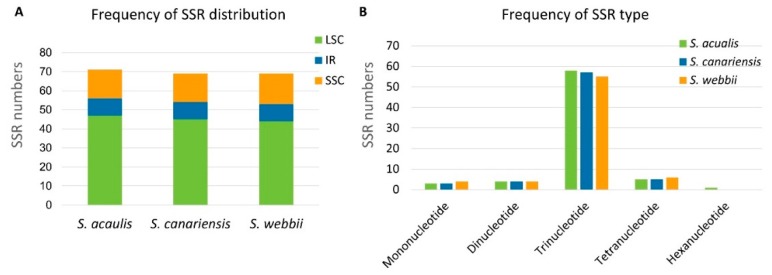
Simple sequence repeat number per distribution and repeat type of three *Sonchus* species. (**A**) Variation in the distribution of SSRs in the chloroplast genomes of each *Sonchus* species. (**B**) Number of SSR motifs in different repeat types of each *Sonchus* species.

In this study, very similar numbers of potential SSRs were identified from the chloroplast genomes of three *Sonchus* species by using MISA [36]: 80 from *S. acaulis,* 78 from *S. canariensis*, and 78 from *S. webbii*. The SSR search parameters were set for 1–15 (mono-nucleotide motifs with 15 minimum numbers of repetition), 2–5, 3–3, 4–3, 5–3, and 6–3. Interestingly, SSRs for all three *Sonchus* species were mainly distributed in the coding regions (61–63%), with much lower quantities distributed in the non-coding introns (4–5%) and intergenic regions (33–34%). Considering the quadripartite regional occupancy of SSRs, the IR and SSC regions were remarkably lower in overall SSR frequency compared with the LSC region: 19–20% from the SSC region and 11–12% from each of the two IR regions versus 56–59% from the LSC region (Figure 4A). However, SSC region occupies the smallest size (12%) in whole chloroplast genome, relatively to LSC (55%) and IR (16%), therefore, SSC region is most enriched in the distribution of SSRs, when taking into account its relative region size. Among the identified SSRs, the tri-nucleotide motifs showed most abundant repeat length (63–66 (81–83%)) with relatively lower proportions of other SSR types (approximately 5–6% of mono-nucleotide, 5% of di-nucleotide, and 6–8% of tetra-nucleotide motifs). There were no penta-nucleotide motifs in all three species. The hexa-nucleotides of *S. acaulis* showed unique characteristics (Figure 4B).

**Figure 5:**
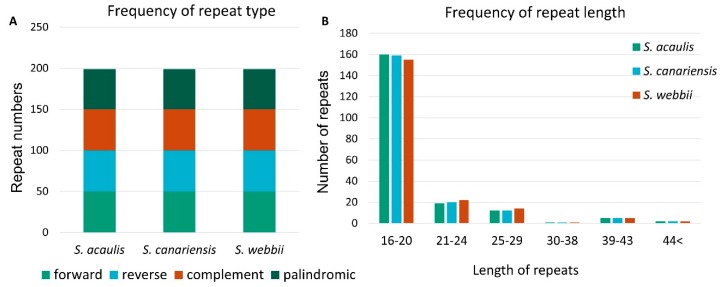
Repeat numbers per repeat type and repeat length of three *Sonchus* species. (**A**) Variation in the distribution of forward, reverse, complement, and palindromic repeats in the chloroplast genomes of each *Sonchus* species. (**B**) Number of different repeat lengths of each *Sonchus* species.

In addition to the SSRs, large repeats on the sequences of the three chloroplast genomes were analyzed using REPuter, considering that the repeated sequences are often associated with the process of genome rearrangement [37]. Using parameters of maximum computed repeats = 50, minimum repeat size = 8 bp, and hamming distance = 0, a total of 199 pairs of repeats containing 50 forward, 50 reverse, 50 complement, and 49 palindromic matches in each *Sonchus* species were identified (Figure 5A). Lengths of 16–20 repeats were the most frequent (78–80%) followed by 21–24 repeats (10–11%) and 25–29 repeats (6–7%), with quite rare numbers of the repeats of over 30 compared with the IRs (Figure 5B). The numbers and distribution patterns of the repeated sequences were remarkably similar and conserved among the three chloroplast genomes. They differed from each other in forward and reverse repeats, while complement and palindromic repeats were identical among them. These species-specific repeat loci found in this study could be used for identification of new genomic regions for use in the phylogenetic studies of *Sonchus* species.

### 3.3. Sequence Divergence and its Hotspots

Analysis of DNA sequence polymorphisms and divergence within and between closely related species can provide insights into the evolutionary forces acting on populations and species. Chloroplast sequence polymorphisms have been extensively used to investigate phylogenetic relationships at wide ranges of taxonomic level in plants. However, reduced and combined data sets of several chloroplast regions often lack enough variation in closely related species, especially those that have diverged recently. The advent of high-throughput sequencing technologies of next-generation sequencing (NGS) has helped reveal considerable genome-wide variations in terms of sequences and structures of entire chloroplast genomes, contributing significantly to the field of chloroplast genetics and genomics [15].

**Figure 6:**
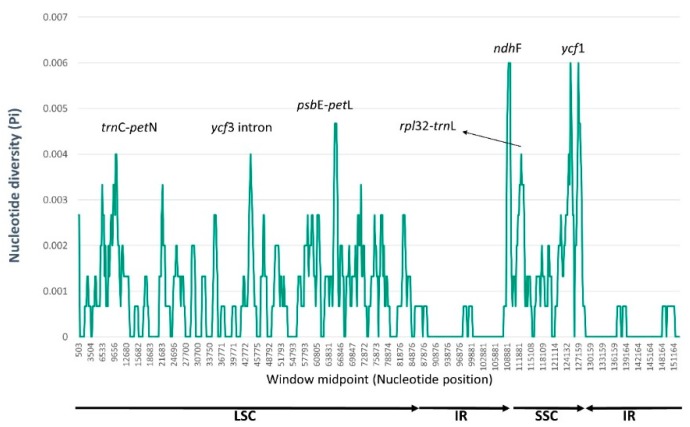
DNA sequence polymorphisms of three *Sonchus* chloroplast genomes calculated using a sliding window analysis of 1000 bases and 200 base step sizes using DnaSP. Six most divergent regions are suggested as divergence hotspots and potential chloroplast markers for *Sonchus* species.

**Figure 7:**
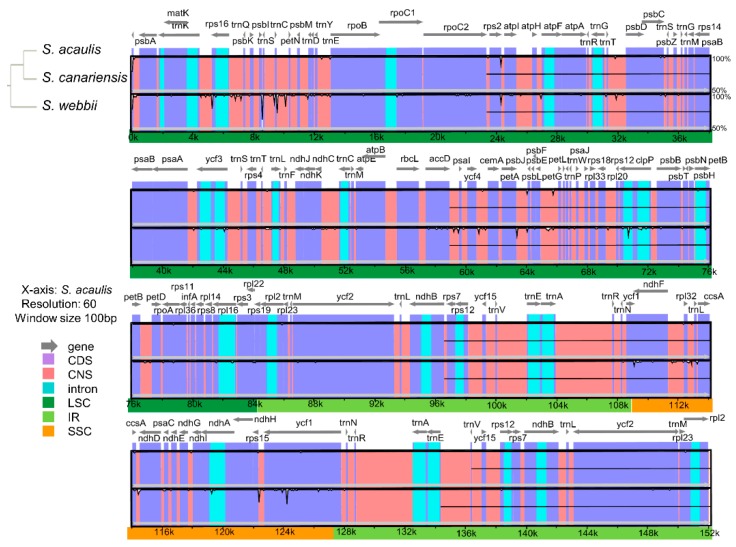
Comparison of the chloroplast genomes of three *Sonchus* species generated by mVISTA; *S. acaulis*, *S. canariensis*, and *S. webbii*. Sequence identity is portrayed with cut-off of 50% identity. The Y-scale axis represents the percent identity within 50–100%. Grey arrows indicate genes with their orientation and position. Genome regions are color-coded as blue blocks for the conserved genes (CDS), pink blocks for the conserved non-coding sequences in intergenic regions (CNS), aqua-blue blocks for introns. Thick lines below the alignment indicate the quadripartite regions of genomes; LSC region is in dark green, IR regions, in light green, and SSC region, in orange. Black bordered white peaks shown in genome regions indicate the divergent regions with sequence variation among three *Sonchus* species.

Based on the NGS analyses performed in this study, nucleotide diversity was calculated using DnaSP with a sliding window analysis (window length = 1000 bp and step size = 200 bp excluding sites with alignment gaps) to estimate the divergence level of different regions in three *Sonchus* species (Figure 6). Overall nucleotide diversity value (Pi) among three chloroplast genomes was 0.00090, ranging from 0 to 0.006. The SSC region showed the highest nucleotide diversity (0.001917) among the regions of LSC, SSC and IRs, while the lowest value was in the IR boundary regions (0.00027). Six divergence hotspots of the most variable regions were suggested as the potential chloroplast markers for phylogenetic studies of *Sonchus* species; three intergenic regions (*trn*C*-pet*N, *psb*E*-pet*L, and *rpl*32*-trn*L), one intron region (*ycf*3 intron), and two protein coding regions (*ndh*F and *ycf*1). Three noncoding regions (*trn*C*-pet*N, *psb*E*-pet*L, and *ycf*3 intron) were located in the LSC region, but two coding regions (*ndh*F and *ycf*1) and one noncoding region (*rpl*32*-trn*L) were located in the SSC region. The result of mVISTA also exhibited a high degree of synteny and gene order conservation across the entire chloroplast genomes of the three *Sonchus* species. A total of 206 polymorphic sites, which were identified in the DnaSP analysis, were visualized in mVISTA graph from mostly noncoding regions, but also from several protein coding regions, such as *rpo*B, *rpo*C2, *atp*A, *acc*D, *psb*C, *rpl*16, *ycf*2, *ndh*F, *ycf*1, and others (Figure 7).

### 3.4. Phylogenetic Analysis

**Figure 8:**
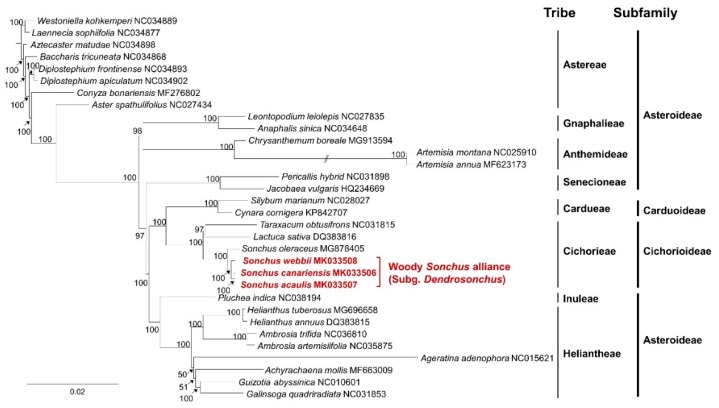
Phylogenetic relationships among 32 species within the family Asteraceae, based on whole chloroplast genome sequences inferred from maximum likelihood analysis by IQ-TREE. Numbers above nodes are bootstrap values with 1000 replicates. The taxonomy of tribe and subfamily levels is presented.

The taxonomic position and evolutionary relationship of three species of the woody *Sonchus* alliance were determined by comparative phylogenetic analysis among 32 representative Asteraceae species based on the relationships of whole chloroplast genomes. The maximum likelihood tree generated by IQ-TREE supported the traditional taxonomy of the family Asteraceae, except the delimitation of the subfamily Asteroideae (Figure 8). The subfamily Asteroideae failed to form a monophyletic clade, supporting the previous study [38]. Two monophyletic tribes of Asteroideae, i.e., Heliantheae and Inuleae, were distantly related to other tribes of the same subfamily. In addition, we found that the tribe Astereae is not monophyletic, while the other tribes of Gnaphalieae, Anthemideae, Senecioneae, Inuleae, and Heliantheae are monophyletic. The genus *Sonchus* was well supported, forming a monophyletic clade including three species sequenced in this study within the tribe Cichorieae of the subfamily Cichoriodeae. The phylogenetic relationship among *Sonchus* species was consistent with previous studies [3,7,8,9,10,11,12]. *Sonchus oleraceus*, an herbaceous annual or biennial weed occurring globally, displayed a sister relationship with the woody *Sonchus* alliance species of the subgenus *Dendrosonchus*. The woody *Sonchus* alliance displayed monophyly, supported strongly by a high bootstrap value, suggesting that it evolved from a common ancestor shared with *S. oleraceus*, probably an herbaceous continental species. Within the woody *Sonchus* alliance, *S. webbii*, which is an herbaceous perennial with tuberous roots, diverged first, followed by the clade containing the woody species *S. acaulis* and *S. canariensis*. The *Sonchus* phylogeny, based on the analysis of the whole chloroplast genome, supported the hypothesis that the herbaceous (annual, biennial, or perennial) habit is plesiomorphic, while the shrub or tree habits of the woody *Sonchus* alliance originated from that of its herbaceous ancestors.

## 4. Conclusions

This study is the first attempt to characterize the chloroplast genomes of the woody *Sonchus* alliance endemic to the Canary Islands and to provide evidence supporting the hypothesis that the origin and evolution of insular endemic species tend towards woodiness on oceanic islands. The results of this study provide rich genetic information in terms of genome sequence differentiation, structure, and mutation hotspots that can be used in evolutionary studies of the woody *Sonchus* alliance, as well as other *Sonchus* species. Comparative genomic analyses revealed that the woody *Sonchus* alliance chloroplast genomes are very conserved, sharing most common genomic features despite the extensive morphological, anatomical, and ecological diversity among three species (*S. acaulis, S. canariensis,* and *S. webbii*). SSRs, large repeat sequences, and highly variable regions of both coding and noncoding regions were identified as potential phylogenetic markers. Phylogenetic relationship based on whole chloroplast genome sequences supported the monophyly of the woody *Sonchus* alliance, suggesting its origin from a single herbaceous continental ancestor followed by adaptive radiation and diversification in situ on the Canary Islands. Owing to limited sampling, the continental progenitor of the woody *Sonchus* alliance remains elusive. Nevertheless, this study provides preliminary data for future studies regarding the origin and evolution of the woody *Sonchus* alliance.

## Figures and Tables

**Table 1:** Summary of the complete chloroplast genome characteristics of three *Sonchus* species in the woody *Sonchus* alliance in the Canary Islands.

Characteristics	*S. acaulis*	*S. canariensis*	*S. webbii*
GenBank accession number	MK033507	MK033506	MK033508
Total cpDNA size (bp)	152,071	152,075	152,194
LSC size (bp)	84,335	84,338	84,269
SSC size (bp)	24,746	24,746	24,749
IR size (bp)	18,244	18,245	18,427
Number of genes	131	131	131
Number of protein-coding genes	88	88	88
Number of tRNA genes	37	37	37
Number of rRNA genes	6	6	6
GC content (frequency/%)	57,252/37.6%	57,252/37.6%	57,227/37.6%
Habit	Woody perennial, erect	Woody perennial, erect	Herbaceous perennial, erect
Height (cm)	100–200	150–300	60–150
Stem	Short and woody	Woody	Thin and pith hollow
Leaf/root characteristics	Basal rosette pinnatifid leaves with acute lobes	Terminal leaf-rosettes on the branches with pinnatisect lobes	Basal rosette variable leaves, pinnatisect to almost entire, long tuberous roots
Habitats on the Canary Islands	Wide spread in forest and xerophytic zones in Tenerife and Gran Canaria	Rare in Tenerife and Gran Canaria	Rare in the N. Coast region in pine forest, La Palma

cpDNA: chloroplast DNA; bp: base pairs; GC: guanine-cytosine.

**Table 2:** Genes present in the complete chloroplast genome of three *Sonchus* species in the woody *Sonchus* alliance on the Canary Islands.

Category	Gene Name
Photosystem I	*psa*A, *psa*B, *psa*C, *psa*I, *psa*J, *ycf*3 ^b^, *ycf*4
Photosystem II	*psb*A, *psb*B, *psb*C, *psb*D, *psb*E, *psb*F, *psb*H, *psb*I, *psb*J, *psb*K, *psb*L, *psb*M, *psb*N, *psb*T, *psb*Z
Cytochrome b6/f complex	*pet*A, *pet*B^a^, *pet*D, *pet*G, *pet*L, *pet*N
Cytochrome C synthesis	*ccs*A
ATP synthase	*atp*A, *atp*B, *atp*E, *atp*F^a^, *atp*H, *atp*I
RuBisCO	*rbc*L
NADH oxidoreductase	*ndh*A^a^, *ndh*B^a,c^, *ndh*C, *ndh*D, *ndh*E, *ndh*F, *ndh*G, *ndh*H, *ndh*I, *ndh*J, *ndh*K
Large subunit ribosomal proteins	*rpl*2 ^a,c^, *rpl*14, *rpl*16 ^a^, *rpl*20, *rpl*22, *rpl*23 ^c^, *rpl*32, *rpl*33, *rpl*36
Small subunit ribosomal proteins	*rps*2, *rps*3, *rps*4, *rps*7 ^c^, *rps*8, *rps*11, *rps*12 ^b,^^c,d^, *rps*14, *rps*15, *rps*16 ^a^, *rps*18, *rps*19
RNA polymerase	*rpo*A, *rpo*B, *rpo*C1 ^a^, *rpo*C2
Translation initiation factor	*inf*A
Others	*acc*D, *cem*A, *clp*P ^b^, *mat*K
Unknown function genes (conserved reading frames)	*ycf*1 ^c^, *ycf* 2 ^c^, *ycf*15 ^c^
Ribosomal RNAs	*rrn*5^c^, *rrn*16^c^, *rrn*23 ^c^
Transfer RNAs	*trn*A-UGC ^a,c^, *trn*C-GCA, *trn*C-ACA ^a^, *trn*D-GUC, *trn*E-UUC, *trn*E-UUC ^a,c^, *trn*F-GAA, *trn*G-GCC, *trn*G-UCC ^a^, *trn*H-GUG, *trn*K-UUU ^a^, *trn*L-CAA ^c^, *trn*L-UAA ^a^, *trn*L-UAG, *trn*M-CAU ^c^, *trn*M-CAU^c^, *trn*N-GUU ^c^, *trn*P-UGG, *trn*Q-UUG, *trn*R-ACG ^c^, *trn*R-UCU, *trn*S-GCU, *trn*S-CGA ^a^, *trn*S-UGA, *trn*S-GGA, *trn*T-UGU, *trn*V-GAC ^c^, *trn*W-CCA, *trn*Y-GUA

^a^ Gene containing a single intron. ^b^ Gene containing two introns. ^c^ Two gene copies in IRs. ^d^ Trans-splicing gene.

## References

[1] Kilian N., Gemeinholzer B., Lack H.W., Funk V.A., Susanna A., Stuessy T.F., Bayer R.J. (2009). Cichorieae. Systematics, Evolution, and Biogeography of Compositae.

[2] Kilian N., Hand R., von Raab-Straube E. Cichorieae Systematics Portal. http://cichorieae.e-taxonomy.net/portal/.

[3] Lee C., Kim S.-C., Lundy K., Santos-Guerra A. (2005). Chloroplast DNA phylogeny of the woody *Sonchus* alliance (Asteraceae: Sonchinae) in the Macaronesian Islands. Am. J. Bot..

[4] Santos-Guerra A. (1990). Evergreen forests in the Macaronesian region. Nature and Environment Series.

[5] Aldridge A.E., Bramwell D. (1979). Evolution within a single genus: *Sonchus* in Macaronesia. Plants and Islands.

[6] Carlquist S. (1974). Island Biology.

[7] Kim S.-C., Crawford D.J., Jansen R.K. (1996). Phylogenetic relationships among the genera of the subtribe Sonchinae (Asteraceae): Evidence from ITS sequences. Syst. Bot..

[8] Kim S.-C., Crawford D.J., Francisco-Ortega J., Santos-Guerra A. (1996). A common origin for woody *Sonchus* and five related genera in the Macaronesian Islands: Molecular evidence for extensive radiation. Proc. Natl. Acad. Sci. USA.

[9] Kim S.-C., Crawford D.J., Jansen R.K., Santos-Guerra A. (1999). The use of a non-coding region of chloroplast DNA in phylogenetic studies of the subtribe Sonchinae (Asteraceae: Lactuceae). Plant Syst. Evol..

[10] Kim S.-C., Crawford D.J., Francisco-Ortega J., Santos-Guerra A. (1999). Adaptive radiation and genetic differentiation in the woody *Sonchus* alliance (Asteraceae: Lactuceae) in the Macaronesian islands. Plant Syst. Evol..

[11] Kim S.-C., Lee C., Mejias J. (2007). A Phylogenetic analysis of chloroplast DNA *mat*K gene and ITS of nrDNA sequences reveals polyphyly of the genus *Sonchus* and new relationships among the subtribe Sonchinae (Asteraceae: Cichorieae). Mol. Phylogenet. Evol..

[12] Kim S.-C., McGowen M.R., Lubinsky P., Barber J.C., Mort M.E., Santos-Guerra A. (2008). Timing and tempo of early and successive adaptive radiations in Macaronesia. PLoS ONE.

[13] Kim S.-C. (2007). Mapping unexplored genomes: A genetic linkage map of the woody *Sonchus* alliance (Asteraceae: Sonchinae) in the Macaronesian Islands. J. Hered..

[14] Kim S.-C. (2012). Mapping unexplored genomes II: Genetic architecture of species differences in the woody *Sonchus* alliance (Asteraceae) in the Macaronesian Islands. J. Plant Res..

[15] Daniell H., Lin C.S., Yu M., Chang W.J. (2016). Chloroplast genomes: Diversity, evolution, and applications in genetic engineering. Genome Biol..

[16] Parks M., Cronn R., Liston A. (2009). Increasing phylogenetic resolution at low taxonomic levels using massively parallel sequencing of chloroplast genomes. BMC Biol..

[17] Wyman S.K., Jansen R.K., Boore J.L. (2004). Automatic annotation of organellar genomes with DOGMA. Bioinformatics.

[18] Laslett D., Canback B. (2004). ARAGORN, a program to detect tRNA genes and tmRNA genes in nucleotide sequences. Nucleic Acids Res..

[19] Lagesen K., Hallin P., Rødland E.A., Stærfeldt H.H., Rognes T., Ussery D.W. (2007). RNammer: Consistent annotation of rRNA genes in genomic sequences. Nucleic Acids Res..

[20] Lohse M., Drechsel O., Kahlau S., Bock R. (2013). OrganellarGenomeDRAW—A suite of tools for generating physical maps of plastid and mitochondrial genomes and visualizing expression data sets. Nucleic Acids Res..

[21] Kurtz S., Choudhuri J.V., Ohlebusch E., Schleiermacher C., Stoye J., Giegerich R. (2001). REPuter: The manifold applications of repeat analysis on a genomic scale. Nucleic Acids Res..

[22] Librado P., Rozas J. (2009). DnaSP v5: A software for comprehensive analysis of DNA polymorphism data. Bioinformatics.

[23] Frazer K.A., Pachter L., Poliakov A., Rubin E.M., Dubchak I. (2004). VISTA: Computational tools for comparative genomics. Nucleic Acids Res..

[24] Brudno M., Do C.B., Cooper G.M., Kim M.F., Davydov E., Green E.D., Sidow A., Batzoglou S. (2003). NISC Comparative Sequencing Program. LAGAN and Multi-LAGAN: Efficient Tools for Large-Scale Multiple Alignment of Genomic DNA. Genome Res..

[25] Katoh K., Standley D.M. (2013). MAFFT multiple sequence alignment software version 7: Improvements in performance and usability. Mol. Biol. Evol..

[26] Nguyen L.T., Schmidt H.A., von Haeseler A., Minh B.Q. (2014). IQ-TREE: A fast and effective stochastic algorithm for estimating maximum-likelihood phylogenies. Mol. Biol. Evol..

[27] Kalyaanamoorthy S., Minh B.Q., Wong T.K., von Haeseler A., Jermiin L.S. (2017). ModelFinder: Fast model selection for accurate phylogenetic estimates. Nat. Methods.

[28] Kim K.J., Choi K.S., Jansen R.K. (2005). Two chloroplast DNA inversions originated simultaneously during the early evolution of the sunflower family (Asteraceae). Mol. Biol. Evol..

[29] Timme R.E., Kuehl J.V., Boore J.L., Jansen R.K. (2007). A comparative analysis of the *Lactuca* and *Helianthus* (Asteraceae) plastid genomes: Identification of divergent regions and categorization of shared repeats. Am. J. Bot..

[30] Wang M.L., Barkley N.A., Jenkins T.M. (2009). Microsatellite markers in plants and insects. Part I: Applications of biotechnology. Genes Genom. Genom..

[31] Powell W., Morgante M., Andre C., McNicol J.W., Machray G.C., Doyle J.J., Tingey S.V., Rafalski J.A. (1995). Hypervariable microsatellites provide a general source of polymorphic DNA markers for the chloroplast genome. Curr. Biol..

[32] Provan J., Soranzo N., Wilson N.J., McNicol J.W., Forrest G.I., Cottrell J., Powell W. (1998). Gene—Pool variation in Caledonian and European Scots pine (*Pinus sylvestris* L.) revealed by chloroplast simple—sequence repeats. Proc. R. Soc. Lond. Biol. Sci..

[33] Ishii T., Mori N., Ogihara Y. (2001). Evaluation of allelic diversity at chloroplast microsatellite loci among common wheat and its ancestral species. Theor. Appl. Genet..

[34] Vendramin G.G., Degen B., Petit R.J., Anzidei M., Madaghiele A., Ziegenhagen B. (1999). High level of variation at *Abies alba* chloroplast microsatellite loci in Europe. Mol. Ecol..

[35] Chung S.M., Staub J.E., Chen J.F. (2006). Molecular phylogeny of *Cucumis* species as revealed by consensus chloroplast SSR marker length and sequence variation. Genome.

[36] Thiel T., Michalek W., Varshney R., Graner A. (2003). Exploiting EST databases for the development and characterization of gene-derived SSR-markers in barley (*Hordeum vulgare* L.). Theor. Appl. Genet..

[37] Palmer J.D., Bogorad L., Vasil I.K. (1991). Plastid chromosomes: Structure and evolution. The Molecular Biology of Plastids.

[38] Wang X.Y., Zhou Z.S., Liu G., Qian Z.Q. (2018). Characterization of the complete chloroplast genome of the invasive weed *Galinsoga quadriradiata* (Asterales: Asteraceae). Conserv. Genet. Resour..

